# Availability and Accessibility of Live Nonreplicating Smallpox/Mpox Vaccine

**DOI:** 10.1001/jamanetworkopen.2023.7873

**Published:** 2023-04-07

**Authors:** Peter A. Kahn, Xiaohan Ying, Michael Virata, Patrick Magahis, Sunnie Li, Walter S. Mathis

**Affiliations:** 1 Section of Pulmonary, Critical Care and Sleep Medicine, Yale School of Medicine, New Haven, Connecticut; 2 Department of Medicine, NewYork Presbyterian–Weill Cornell, New York, New York; 3 Section of Infectious Diseases, Yale School of Medicine, New Haven, Connecticut; 4 Department of Medicine, Weill Cornell Medicine, New York, New York; 5 University of North Carolina at Chapel Hill; 6 Department of Psychiatry, Yale School of Medicine, New Haven, Connecticut

## Abstract

This cross-sectional study compares the availability and accessibility of mpox vaccine sites with the number of reported cases and allocated vaccines.

## Introduction

On July 23, 2022, the World Health Organization declared monkeypox (mpox) a public health emergency of international concern, and on August 4, 2022, the US Department of Health and Human Services followed suit. Five days later, the US Food and Drug Administration authorized emergency use of the smallpox/mpox vaccine Jynneos (Bavarian Nordic) in an effort to contain this outbreak. With a severely constrained supply, the vaccine has been distributed by the Centers for Disease Control and Preventionto states’ departments of public health.

Given this centralized distribution, there were concerns about equitable access to the vaccine.^1,2^ Notably, the accessibility of vaccine sites was a major obstacle for many who wished to be vaccinated. In this cross-sectional study, we therefore sought to understand the availability and accessibility of vaccine administration sites, compared with the number of reported cases and allocated vaccines.

## Methods

This study was not submitted for institutional review board approval and did not require informed consent because it used aggregate and deidentified public data and was not human participant research as defined by 45 CFR §46. We followed the Strengthening the Reporting of Observational Studies in Epidemiology (STROBE) reporting guideline.

Locations of vaccination sites were obtained from individual state government websites during the week of August 1 to August 5, 2022. Each state’s Department of Public Health was then contacted via telephone to confirm the vaccination sites. Case incidence data were collected from the Centers for Disease Control and Prevention and state websites, and vaccine allocation data were collected from the US Department of Health and Human Services.^3,4,5^ Population, race and ethnicity, and geographic data were collected from the 2020 US Census.^6^ Minimum travel time to vaccination site was computed using a server from Open Source Routing Machine, which was built on OpenStreetMap road network data. Spearman rank correlation (ρ) was computed to determine whether the number of vaccines shipped and number of vaccine sites were correlated with the number of mpox cases. The Spearman rank correlation tests were run as two-sided and a *P* value of 0.05 was used to infer statistical significance. R statistical software version 4.2.1 (R Project for Statistical Computing) was used to conduct the analysis.

## Results

By August 5, 2022, there were 247 designated vaccination sites in the 26 states and Washington, DC. The remaining 24 states distributed vaccines on a case-by-case basis (Table). The number of cases was highly correlated with the number of vaccines shipped (ρ = 0.95; *P* < .001) but was less correlated with number of vaccine sites (ρ = 0.63; *P* < .001) (Table).

**Table.** Number of Cases, Shipped Vaccines, and Vaccination Sites, for 10 States With Most Cases in August 2022

State	Mpox cases, No.	Vaccines shipped, No.	Vaccine sites, No.	Mpox cases, %^a^					Population, No. (%)									
									≤30 min drive from vaccine site					>60 min drive from vaccine site				
				AIAN	Asian	Black	Hispanic	White	AIAN	Asian	Black	Hispanic	White	AIAN	Asian	Black	Hispanic	White
New York	1862	94 259	27	0	3.9	28.7	35.6	30.1	23 698 (0.2)	1 529 769 (10.3)	2 446 083 (16.5)	3 363 402 (22.7)	6 438 872 (43.4)	12 156 (0.4)	67 169 (2.2)	141 247 (4.6)	139 342 (4.6)	2 590 877 (84.7)
California	826	109 531	28	0	6.8	12.1	39.1	38.2	26 708 (0.2)	2 388 722 (17.8)	980 871 (7.3)	5 750 689 (42.9)	3 785 965 (28.2)	89 908 (0.5)	1 736 277 (9.2)	863 929 (4.6)	7 402 647 (39.4)	7 761 271 (41.3)
Florida	633	65 960	2	NA	NA	NA	NA	NA	4115 (0.1)	102 414 (2.7)	689 639 (18.5)	2 019 974 (54.1)	816 377 (21.9)	29 012 (0.2)	348 736 (2.5)	1 830 002 (13.3)	2 121 750 (15.4)	8 871 967 (64.4)
Texas	606	44 420	20	NA	NA	NA	NA	NA	28 655 (0.2)	1 041 748 (7.2)	2 090 750 (14.4)	5 841 695 (40.3)	4 956 243 (34.2)	24 018 (0.3)	124 166 (1.4)	713645 (8.0)	3 829 512 (42.8)	4 084 237 (45.7)
Illinois	602	46 207	12	NA	NA	NA	NA	NA	4063 (0.1)	321 331 (6.5)	1 151 952 (23.4)	1 226 111 (24.9)	2 016 990 (41.0)	5761 (0.1)	86 139 (2.1)	284 235 (6.9)	289 336 (7.0)	3 395 066 (82.0)
Georgia	596	21 782	5	0.2	0.8	80.5	7.7	13.8	5950 (0.1)	289 416 (7.2)	1 530 204 (38.1)	503 066 (12.5)	1 473 171 (36.7)	8200 (0.2)	50 685 (1.3)	1 106 918 (27.5)	287 378 (7.2)	2 432 391 (60.5)
Washington, DC	283	21 755	3	0.3	2.5	35.5	17.7	41.9	1298 (0.2)	28 347 (4.1)	312 661 (45.3)	77 981 (11.3)	257 792 (37.4)	0	0	0	0	0
Pennsylvania	205	11 501	11	NA	NA	NA	NA	NA	5575 (0.1)	293 201 (6.4)	1 002 594 (21.7)	354 283 (7.7)	2 710 896 (58.8)	5572 (0.1)	107 321 (1.7)	271 463 (4.4)	460 700 (7.5)	5 121 625 (83.3)
New Jersey	188	11 329	5	0	4.6	23.0	40.2	28.7	5528 (0.1)	528 040 (9.1)	807 432 (13.9)	1 331 087(22.8)	2 772 507 (46.7)	1614 (0.2)	33 728 (5.1)	55 923 (8.5)	75 666 (11.5)	458 407 (69.4)
Washington	175	6930	2	NA	NA	NA	NA	NA	8110 (0.4)	378 743 (16.8)	125 143 (5.5)	225 061 (10.0)	1 328 678 (58.9)	49 737 (1.4)	91 889 (2.7)	58 313 (1.7)	543 830 (15.7)	2 449 654 (71.0)
Other states with vaccine sites^b^	924	92 190	132	NA	NA	NA	NA	NA	199 663 (0.5)	2 053 989 (4.9)	6 726 236 (15.9)	6 145 149 (14.5)	25 244 114 (59.7)	383 947 (1.3)	480 133 (1.6)	2 312 218 (7.7)	2 526 004 (8.4)	23 316 021 (77.5)
Nationwide	7488	577 826	247	0	3	36	31	27	320 892 (0.3)	9 307 741 (8.1)	18 776 162 (16.3)	27 505 229 (24.0)	53 379 142 (46.5)	1 419 581 (0.9)	4 450 985 (2.9)	14 731 679 (9.5)	22 136 487 (14.3)	105 596 022 (68.3)

Abbreviations: AIAN, American Indian and Alaska Native; mpox, monkeypox; NA, not applicable.

^a^ Percentages in these columns come from Ndugga et al.^3^

^b^ Other states with vaccine sites included Arkansas, Arizona, Colorado, Connecticut, Louisiana, Massachusetts, Michigan, Missouri, Nevada, North Carolina, North Dakota, Ohio, Oregon, Rhode Island, South Carolina, Utah, and Wisconsin.

In the US, 17.1% (56 169 661) of the population lived within 15 minutes of a vaccination site, 17.8% (58 709 295) lived 15 to 30 minutes from a site, and 47% (154 594 092) lived more than an hour away from the nearest vaccination site. California had the largest number of sites (28 sites), but Connecticut, Rhode Island, and Washington, DC, were the only areas where the entire population lived within 45 minutes of a site (Figure). Nationally, 46.5% of White people (53 379 142) lived within 30 minutes of the nearest vaccination site, compared with 16.3% (18 776 162) of Black people and 24.0% (27 505 229) of Hispanic people. In the 5 states with the highest number of mpox cases, Black people consistently had worse geographic access compared with White and Hispanic people.

**Figure.**
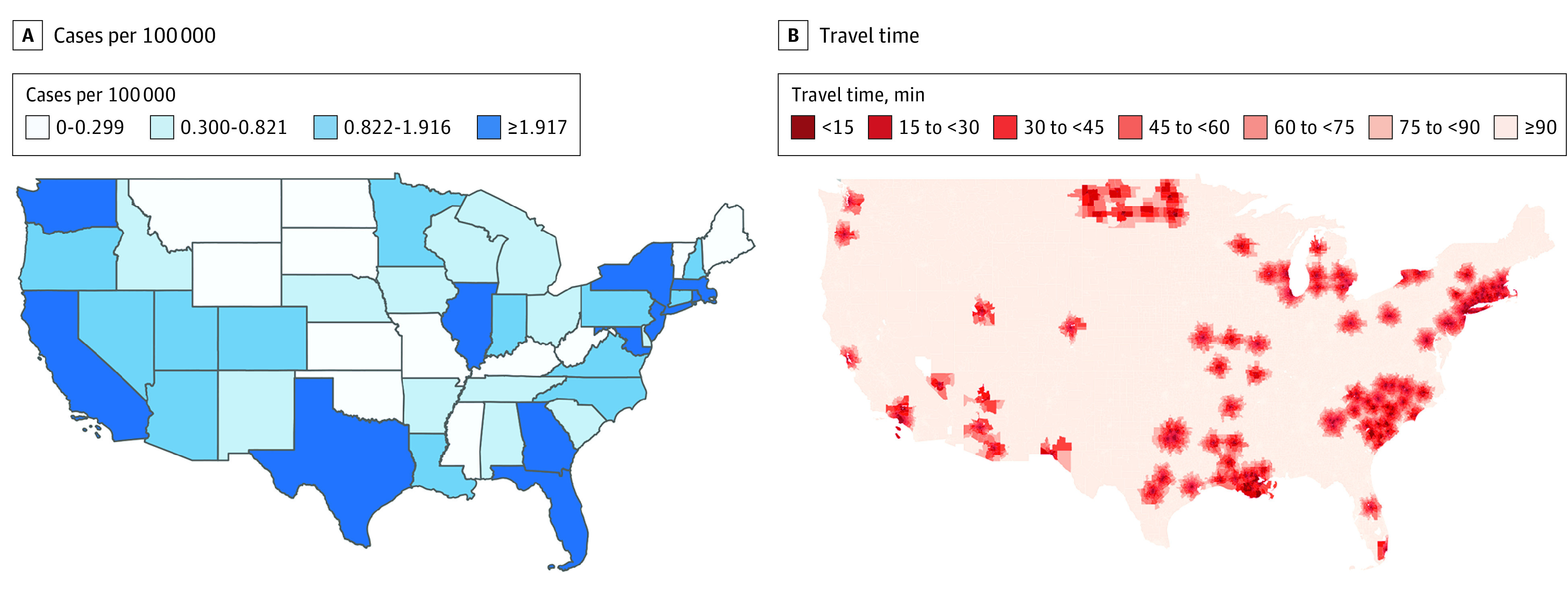
State Prevalence Rate and Minimum Travel Time to Mpox Vaccination Site as of August 2022 Maps show US cases of mpox by state per 100 000 (A) and travel time to a destination site (B).

## Discussion

Despite the importance of vaccination, this cross-sectional study revealed that close to one-half of the US population lived more than an hour away from vaccination sites. Although some states effectively ensured access across the entire state, they were outliers. We found that there were significant racial disparities in mpox vaccine access across the US. Additionally, the number of vaccines shipped correlated with the number of mpox cases, but the number of vaccination sites did not, further raising concerns about vaccine accessibility.

Our study has limitations. Travel time calculations did not incorporate traffic patterns or public transit options. With the dynamic supply of the vaccine, including recent changes to include subcutaneous administration, vaccine sites are expected to shift over time. The presence of a vaccination site does not guarantee availability of the vaccine. State and national policy has also changed in response to shifting public health conditions since the time this study was conducted.

Although the supply of the mpox vaccine has been heavily constrained, additional attention to ensuring equity in access is needed. At the time this study was conducted, one-half of states had yet to establish mpox vaccine clinics despite known cases of mpox in 48 states. Among states that already have vaccine clinics, almost one-half of the population lived more than an hour away from a vaccination site. As new data become available, federal, state, and local policy makers should work to ensure that the vaccine is accessible to as much of the population as possible.

## Notes

Supplement.
Data Sharing Statement
