# Supplementary material for: Availability and Accessibility of Live Nonreplicating Smallpox/Mpox Vaccine
Source: JAMA Netw Open. 2023 Apr 7;6(4):e237873. doi: 10.1001/jamanetworkopen.2023.7873 (PMC10082399; doi:10.1001/jamanetworkopen.2023.7873)
Supplement: Supplement. — Data Sharing Statement [file jamanetwopen-e237873-s001.pdf]

Data Sharing Statement

Kahn. Availability and Accessibility of Live Nonreplicating Smallpox/Mpox Vaccine. *JAMA Netw Open*. Published April 07, 2023. doi:10.1001/jamanetworkopen.2023.7873

Data
Data available: Yes
Data types: Data (not involving human participants)
How to access data: walter.mathis@yale.edu
When available: With publication

Supporting Documents
Document types: None

Additional Information
Who can access the data: anyone requesting data
Types of analyses: for any purpose
Mechanisms of data availability: with investigator support
